# The recurrent *WASF1* nonsense variant identified in two unaffected Chinese families with neurodevelopmental disorder: case report and review of the literatures

**DOI:** 10.1186/s12920-023-01630-8

**Published:** 2023-08-28

**Authors:** Xiang Tang, Guoqing Liu, Li Lin, Nong Xiao, Yuxia Chen

**Affiliations:** 1 Department of Rehabilitation, Children’s Hospital of Chongqing Medical University, Chongqing, P.R. China; 2 Ministry of Education Key Laboratory of Child Development and Disorders, Chongqing, P.R. China; 3 National Clinical Research Center for Child Health and Disorders, Chongqing, P.R. China; 4 China International Science and Technology Cooperation base of Child development and Critical Disorders, Chongqing, P.R. China; 5 Children’s Hospital of Chongqing Medical University, Chongqing, P.R. China; 6 Chongqing Key Laboratory of Pediatric, Chongqing, P.R. China

**Keywords:** WASF1 gene, Developmental delay, Absent language, Whole-exome sequencing, Case report

## Abstract

**Background:**

Neurodevelopmental disorder with absent language and variable seizures (NEDALVS, # 618707) are characterized by delayed speech and motor development, ocular abnormalities, and seizures. NEDAVLS is an autosomal dominant disorder caused by de novo mutations in the wasp protein family member 1 (WASF1) gene.

**Case presentation:**

We identified a de novo nonsense variant c.1516 C > T (p.Arg506*) of WASF1 gene (NM_003931.3) in two pediatric female patients with delayed motor and language development.

**Conclusion:**

This case demonstrates the effective role of WES in the diagnosis of NEDALVS. To the best of our knowledge, this variant has not been reported in the Chinese population. This contributes to our further understanding of the disease and to research related to the genetic and clinical heterogeneity, the treatment and prognosis of the disease.

## Background

Neurodevelopmental disorder with absent language and variable seizures (NEDALVS, # 618707) is an autosomal dominant disorder of neurodevelopment, which has clinical heterogeneity [1]. The phenotype of NEDALVS involves multiple organs and body parts [2]. Nervous system phenotypes include intellectual impairment, motor developmental delay, unsteady gait, language deficits, autistic features, and seizures. NEDALVS also cause strabismus, exophthalmia, gray sclera, deep-set eyes, oblique palpebral fissures. Some patients also have abnormalities in the chest, abdomen, or bones [3].

The Wiskott-Aldrich homology 2 or WASP-homology 2 (WH2) domain is an evolutionarily conserved sequence motif in proteins [4]. It is present in WASP proteins that control actin aggregation, so WH2 is important in cellular processes such as contraction, motility, cell transport and cell signaling [5].

## Methods

### Case presentation

**Fig. 1:**
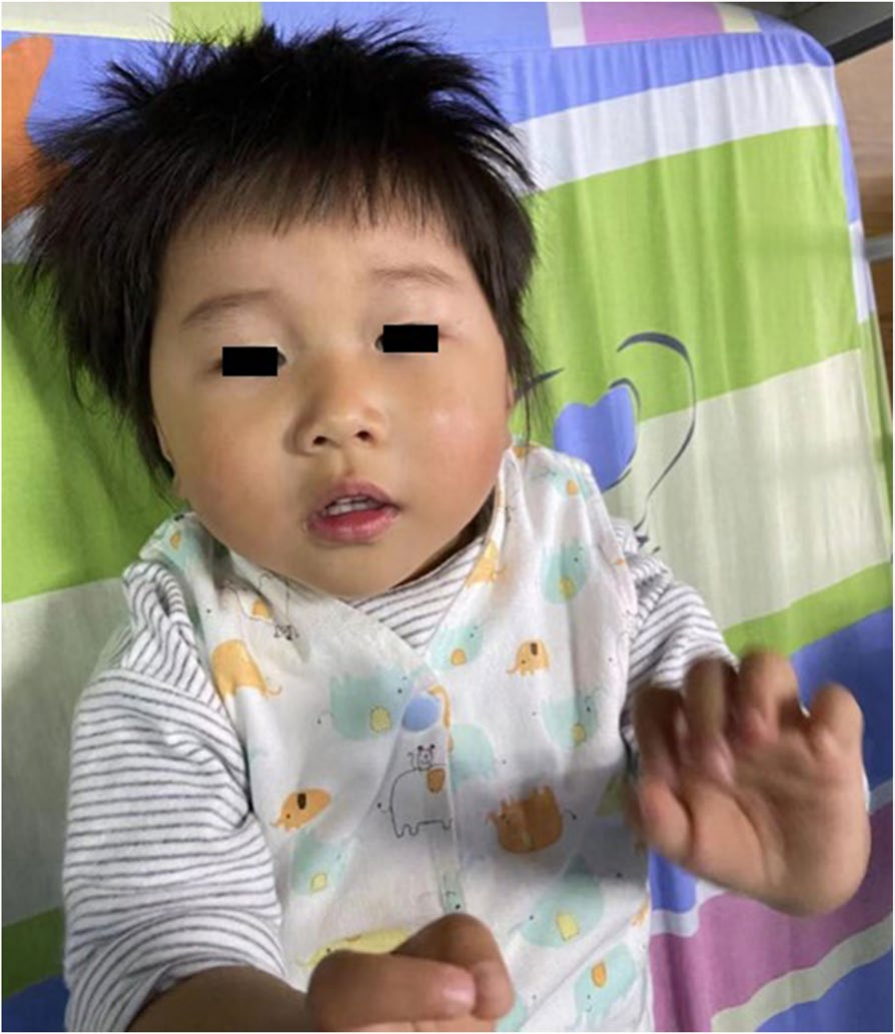
The picture of patient 1

Patient 1 was a girl at 2 years and 4 months old (Fig. 1). She was admitted to hospital, with delayed motor, language and cognitive development. At first, the child falls behind mainly in language and cognition. With age, the movement ability improved than before, and there was no developmental regression. The child can walk independently, but cannot run or jump, hold objects with his fingers, or recognize facial features. The child can occasionally perform simple verbal commands, imitate simple movements, wave goodbye, clap hands. The girl has an exotropia in her right eye. She had a slight decrease in muscle tone in both lower limbs, symmetrical knee reflexes and negative Barthel sign. The patient was negative for ankle clonus. In addition, the child has thick, pale yellow hair with a wide back hairline, a 0.5 × 3 cm melanoma on the left jaw, and a large green spot on the back. The mother suffered from hypothyroidism during pregnancy and was given oral euthalazine. The mother had no history of epilepsy. Tests in other hospitals showed that the first EEG showed increased theta activity during the waking background period; Later, the EEG was normal. Head MRI performed in other hospitals indicated mild widening of the left extratemporal space and left ventricle, abnormal signals in the right cerebellar hemisphere, and low signals on DWI. The Gesell intelligence development test showed that the child had developmental delays, language and motor delays. The child was not considered cerebral palsy and we made a diagnosis of total developmental delay (Table 1).

**Table 1:** Clinical features of patients with the de novo heterozygous variant of WASF1 c.1516 (p.Arg506*) in previously reports and this report

Patient	Author; years	Sex	Age at last exam	Country	Seizure	Neurological (intellectual, motor and language development, etc.)	Craniofacial	Other
1	Ito et al.; 2018 [3]	male	21 years	Canada	yes, onset at 8 years; focalwith occasional GTC	intellectual disability (severe to profound); hypotonia; single words; wide-based gait with poor balance; high pain tolerance	midface hypoplasia; deep set, strabismus, gray sclera	joint hyperflexibility; ankle valgus; long tapered fingers; narrow, pes planus, short forth toes; widely spaced nipples; cafe´ au lait macules; trouble sucking, reflux, easy choking; constipation
2	Ito et al.; 2018 [3]	male	23 years	France	yes, onset at 6 years; absence and GTC	intellectual disability (moderate to severe); hypotonia; simple sentences	midface hypoplasia; exophthalmia	short third toes
3	Ito et al.; 2018 [3]	male	23 years	France	no	intellectual disability (moderate to severe); no seizures; hypotonia; single words; wide-based gait with poor balance; high pain tolerance	strabismus	joint hyperflexibility; knee recurvatum; pes planus; feeding difficulties, reflux; constipation
4	Srivastava et al.;2021 [6]	male	7 years	NA	yes, onset NA; focal seizures, reflex seizures	intellectual disability (moderate); seizure; axial hypotonia; no words; head banging, hitting	long face, simple ears; Strabismus, exotropia	pes planus; constipation
5	Yamamoto et al. 2021 [2]	female	6 years	Japan	yes, onset NA	intellectual disability (severe);	midface hypoplasia; strabismus	ankle valgus; pes planus; feeding problems
6	this report	female	2 year and 4 months	China	no	global developmental delay; motor developmental delay; cognitive impairment; delayed speech and language development; hypotonia	abnormal fundus examination	abnormality of the posterior hairline; melanocytic nevus; thick hair
7	this report	female	1 year and 4 months	China	no	global developmental delay; motor developmental delay; cognitive impairment; delayed speech and language development; no obvious abnormality in muscle tone	strabismus	N/A

Patient 2 was a girl at 1 year and 4 months old. She was admitted to the hospital due to the discovery of motor, language, and intellectual development delay. The child had motor retardation, accompanied by language and cognitive retardation, but no abnormal posture, no definite seizures, and no developmental regression. The child had repetitive stereotypes, such as eating and clapping her hands. Physical examination showed no abnormality. The girl was suspected hypertrophy of the gastrocnemius muscles of both lower extremities. She could execute simple commands, crawl, stand alone, and walk with her head back. Her grip was inflexible. There was no asphyxia at birth, and no abnormality was reported during pregnancy. She was no history of seizures. The borderline EEG suggested that the background occipital region was not dominant during wakefulness with increased 3–5 Hz delta theta mixed activity. The re-examination of the EEG showed that the boundary EEG showed excessive low-amplitude beta activity in the whole brain during wakefulness. Brain MRI showed that the posterior horns and body of the bilateral ventricles were slightly widened. The patient underwent developmental milestone assessment in our hospital. The child underwent the GMFM (88 items) gross motor, FMFM (88 items) fine motor Assessment and Gesell intelligence development test. The tests showed that the child had intellectual developmental delays and speech development delays (Table 1).

### Trio-WES/CNVseq and bioinformatics analysis

After obtaining parental consent and signing informed consent, blood collection was carried out. 3ml of anticoagulant blood was extracted from the venous blood of the children and parents, and trio whole exome sequencing (WES) and low depth whole genome sequencing (CNVseq) were performed. The sequencing was conducted with the xGen Exome Research Panel v2.0 chip through Illumina NovaSeq 6000 series sequencer (PE150). The coverage of target sequence sequencing was not less than 99%. The CNVseq resolution could reach 100 kb. Bioinformatics annotation of detected variants based on public process databases such as ClinVar, HGMD, gnomAD, Exome Sequencing Project and 1000 Genomes Project and population frequency databases It is also predicted to have pathogenic or harmful effects based on the data in the database. The genetic disease precision diagnosis Cloud platform system (Chigene), which integrates molecular biological annotation, biological, genetic and clinical characteristics analysis, was used to analyze and screen hundreds of thousands of gene variants. The classification of variation mainly refers to the three-element classification system and the gene variation classification system of ACMG Classification Standards and Guidelines for Genetic Variation in 2015 [7].

### Molecular results

**Fig. 2:**
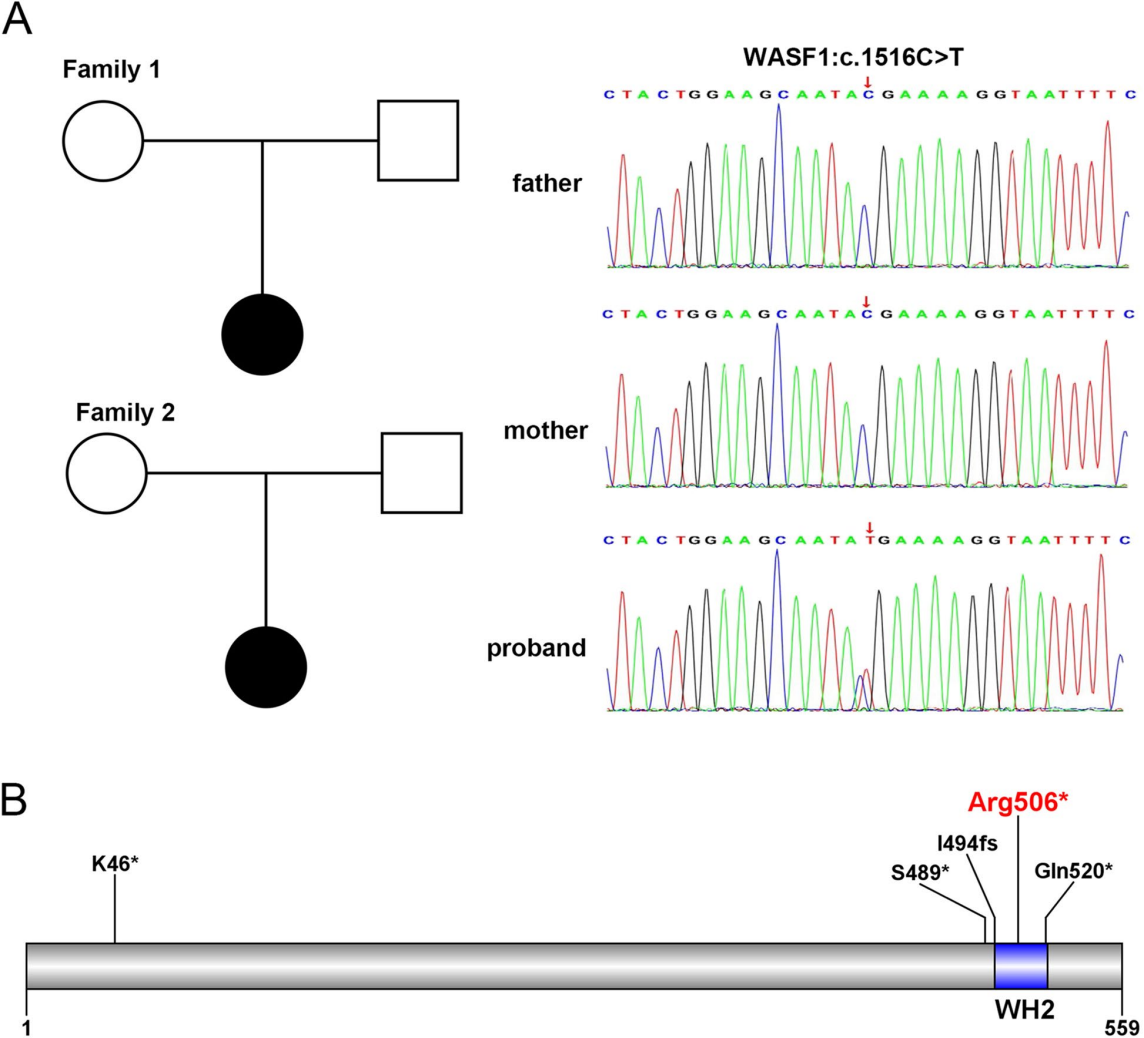
**A** The pedigree of the two families. The de novo variant WASF1:c.1516 C > T is identified in two Chinese families respectively. **B** Distribution of truncation variants included in HGMD database in WASF1 protein domain pattern

The CNVseq results did not reveal suspiciously large fragment CNV deletions or duplications. By trio-whole exome sequencing (trio-WES), a heterozygous nonsense variant of the WASF1 gene c.1516 C > T (p.Arg506*) was identified in two female patients from Chinese families respectively. Both families have wild-type parents, indicating that the mutation is *de novo* (Fig. 2A). This variant is pathogenic (PVS1 + PS2 + PM2) according to ACMG guidelines [7]. This variant is a LOF variant that leads to possible loss of gene function (PVS1); It is a parent-verified de novo variant through pedigree verification (PS2); This site has a frequency of less than 0.0005 in all normal population databases (PM2).

The WASF1 protein is 559 amino acid residues long, of which the 494–521 region is an important functional domain, WH2 (Wiskott-Aldrich homology 2, or WASP-homology 2) (Fig. 2B). Currently, only 4 truncating mutations in the WASF1 gene are included in the HGMD database. Currently, only 4 truncating mutations in the WASF1 gene are included in the HGMD database. Among them, three variants are located in the WH2 functional domain, including c.1516 C > T(p.Arg506*) (Fig. 2B).

## Discussion

We identified two Chinese patients with WASF1:c.1516 C > T(p.Arg506*). Proband 1 (2 years old, female) had delayed language development, cognitive impairment, hypotonia, and left eye esotropia. Proband 2 (1 year old, female) had esotropia in the right eye, unsteady walking, and delayed motor development (Table 1). To the best of our knowledge, this variant locus has not been reported in the Chinese population.

At present, few cases of diseases caused by WASF1 gene variants have been reported. In 2018, Ito et al. [3] reported five unrelated adult patients with similar neurodevelopmental abnormalities ranging in age from 21 to 30 years old. These patients had identified WASF1 variants. Three (age from 21 to 23) of them had the nonsense mutation c.1516 C > T(p.506*) (Table 1). Another nonsense variant c.1558 C > T(p.Gln520*) was tested in 1 individual. A frameshift variant c.1482delinsGCCAGG(p.Ile494Metfs*23) was found in 1 individual. The three patients with the c.1516 C > T variant were all intellectually disabled, hypotonia, impaired speech development, and less unstable. Their eyes were abnormal, such as strabismus, exophthalmia. Two of the three patients had epilepsy and the other had no seizures. Srivastava et al. [6] also reported in 2021 a 7-year-old male patient with the WASF1:c.1516 C > T mutation who was intellectually disabled; seizures; axial hypotonia; speechless; head banging, hitting people; long face, simple ears; strabismus, exotropia (Table 1). According to the above literature review, there were four cases about WASF1: c.1516 C > T(p.Arg506*) in history, and all patients were adult males. The main phenotypic features of these patients are intellectual disability, delayed language and motor development, epilepsy, hypotonia, and ocular abnormalities. We report two female children with this variant. The main clinical features are also phenotypic ocular abnormalities, hypotonia and bradykinesia, but no seizures have been observed. This may be because of young age, epilepsy phenotype has not been fully manifested. Based on the findings of the above cases, the variant c.1516 C. T(p.Arg506*) may be a mutation hotspot of WASF1 gene.

The variant WASF1: c.1516 C > T(p.Arg506*) is located in the WH2 functional domain (Fig. 2B), which resulted in the truncation of 53 amino acid residues in the mutant protein, destroying the key functional domain WH2 (Wiskott-Aldrich homology 2, or WASP-homology 2). The WH2 domain is an actin-binding motif. This domain was first recognized by the mammalian Wiskott-Aldrich syndrome protein (WASP) family as an essential element for regulating the cytoskeleton. WH2 proteins are found in eukaryotes from yeast to mammals, insect viruses and some bacteria. The WH2 domain was found to be a modular part of a larger protein. It can be associated with WH1 or EVH1 domain INTERPRO and CRIB domain INTERPRO, and the WH2 domain can appear as a tandem repeat. The WH2 domain binds actin monomers and can facilitate the assembly of actin monomers into newly formed actin filaments [8, 9].

Some known proteins contain WH2 domains. Baker’s yeast Verprolin, a protein involved in cytoskeletal organization and cell growth [10]. Mammalian WASP, possibly a regulator of lymphocyte and platelet function. Defects in WASP are the cause of Wiskott-Aldrich syndrome (WAS), an X-linked recessive disorder characterized by immune dysregulation and micro thrombocytopenia [11, 12]. WASP protein binds the actin nucleation protein complex Arp2/3 [13]. Mammalian N-WASP/WASL and WASF/SCAR/WAVE1-3, and yeast LAS17, which are also proteins of the WASP family, are involved in signaling from the cell surface to the actin cytoskeleton [14–16]. WAS protein family homolog 1 (WASH1), acts as a nucleation-promoting factor at the endosomal surface, where it recruits and activates the Arp2/3 complex to induce actin polymerization [17]. The WH2 domain plays an important role in a variety of proteins. Once it is destroyed, the protein function will be damaged, which will affect or impair a variety of normal biological activities.

## Conclusions

In conclusion, we detected the WASF1:c.1516 C > T (p.506*) in two Chinese girls by trio-WES, which confirmed that they suffered from Neurodevelopmental disorder with absent language and variable seizures ((NEDALVS, # 618707). This mutation could be a mutation hotspot of WASF1 gene and was located in an important functional domain. In both cases, the molecular pathogenicity was detected by WES at an early age. This highlighted the important role of WES in the early diagnosis of rare diseases and provided a clear direction for possible therapeutic outcomes. As little has been reported about NEDALVS, our report provided new material and information for further understanding of this disease.

## Data Availability

The datasets generated during and/or analysed during the current study are available from the corresponding author on reasonable request. And the variant has been submitted to ClinVar under Accession: SCV002761343.1 (https://www.ncbi.nlm.nih.gov/clinvar/variation/561980/).
